# Metabolic Induction and Early Responses of Mouse Blastocyst Developmental Programming following Maternal Low Protein Diet Affecting Life-Long Health

**DOI:** 10.1371/journal.pone.0052791

**Published:** 2012-12-27

**Authors:** Judith J. Eckert, Richard Porter, Adam J. Watkins, Elizabeth Burt, Suzanne Brooks, Henry J. Leese, Peter G. Humpherson, Iain T. Cameron, Tom P. Fleming

**Affiliations:** 1 Faculty of Medicine, University of Southampton, Southampton General Hospital, Southampton, United Kingdom; 2 Centre for Biological Sciences, University of Southampton, Southampton General Hospital, Southampton, United Kingdom; 3 Centre for Cardiovascular and Metabolic Research, The Hull York Medical School, University of Hull, Hull, United Kingdom; 4 Department of Biology, University of York, York, United Kingdom; Michigan State University, United States of America

## Abstract

Previously, we have shown that a maternal low protein diet, fed exclusively during the preimplantation period of mouse development (Emb-LPD), is sufficient to induce by the blastocyst stage a compensatory growth phenotype in late gestation and postnatally, correlating with increased risk of adult onset cardiovascular disease and behavioural dysfunction. Here, we examine mechanisms of induction of maternal Emb-LPD programming and early compensatory responses by the embryo. Emb-LPD induced changes in maternal serum metabolites at the time of blastocyst formation (E3.5), notably reduced insulin and increased glucose, together with reduced levels of free amino acids (AAs) including branched chain AAs leucine, isoleucine and valine. Emb-LPD also caused reduction in the branched chain AAs within uterine fluid at the blastocyst stage. These maternal changes coincided with an altered content of blastocyst AAs and reduced mTORC1 signalling within blastocysts evident in reduced phosphorylation of effector S6 ribosomal protein and its ratio to total S6 protein but no change in effector 4E-BP1 phosphorylated and total pools. These changes were accompanied by increased proliferation of blastocyst trophectoderm and total cells and subsequent increased spreading of trophoblast cells in blastocyst outgrowths. We propose that induction of metabolic programming following Emb-LPD is achieved through mTORC1signalling which acts as a sensor for preimplantation embryos to detect maternal nutrient levels via branched chain AAs and/or insulin availability. Moreover, this induction step associates with changes in extra-embryonic trophectoderm behaviour occurring as early compensatory responses leading to later nutrient recovery.

## Introduction

Periconceptional environment has been shown to influence the subsequent programme of development in animal models with long-lasting consequences affecting the physiology and health status of offspring [1]–[5]. Thus, in rodents, culture conditions in vitro [6]–[13] and maternal dietary protein restriction in vivo [14]–[17] during preimplantation development are associated with abnormal postnatal growth and increased adult-onset disease risk, characterized particularly in cardiovascular, metabolic and behavioural dysfunction. Similar phenomena have also been observed in related ruminant embryo models [18]–[21]. Collectively, these environmental sensitivities of early developmental stages provide support for an important component of the ‘Developmental Origins of Health and Disease’ (DOHaD) hypothesis, derived from epidemiological and experimental datasets, proposing increased susceptibility to chronic diseases in later life from a deprived in utero experience [22]–[25]. Embryo environment effects also have implications for the safety of assisted conception practices [26]–[29].

Our recent work using the mouse protein restriction model has demonstrated that mothers fed a low protein diet (9% casein) exclusively during the preimplantation period (E0-3.5; Emb-LPD) generated offspring displaying life-long hypertension compared with controls, and associated with attenuated arterial vasodilatation, elevated lung angiotensin-converting enzyme activity, hyperactive behavior and increased adiposity [15]–[17]. Our data also indicated that an early response to this transient dietary challenge (or following LPD maintained throughout gestation) concerned activation of compensatory mechanisms to stabilize later growth of the conceptus. This included evidence of enhanced maternal-fetal nutrient transport capacity via the visceral yolk sac in late gestation and a corresponding increase both in conceptus and birth weight following maternal Emb-LPD [15]. Moreover, the early induction of growth compensation appeared critical in the later onset of disease since Emb-LPD offspring perinatal weight correlated positively with adult weight and susceptibility to cardiovascular and behavioural disease [15].

The current study is concerned with the mechanisms of induction of adverse developmental programming using this model and the early steps in compensatory responses. We believe induction has occurred by the blastocyst stage since transfer of blastocysts collected from Emb-LPD mothers into recipients fed control diet before and subsequent to transfer exhibit the characteristic enhanced growth phenotype in late gestation [15]. Past studies have identified a plethora of communication processes operate within the post-conception maternal tract regulating embryo developmental progression and differentiation, immune system modulation and synchronization of implantation, commonly mediated by a range of steroid hormones, growth factors and cytokines [30]–[35]. Whilst none of these signalling mechanisms can be excluded from a role in ‘nutrient sensing’ by the embryo, we anticipate that the key factors involved are directly growth regulatory given the association between conceptus growth and later disease discussed above. We focus on the potential role of insulin and amino acid (AA) levels as signaling components involved in nutrient sensing. Both of these factors act to control proliferation and cellular phenotype during early development with long-lasting positive effects on fetal growth [36]–[42]. Insulin and the branched chain AAs, especially leucine, also contribute to cellular growth though intracellular mTORC1 signalling [43]–[45]. Moreover, mTORC1 has previously been implicated a role in mouse blastocyst trophoblast motility [38], [40].

We have evaluated the effect of Emb-LPD treatment on mouse maternal systemic and reproductive tract physiology and embryo development and phenotype up to the time of implantation. In particular, we focus on dietary effects on AA availability within the maternal circulation and uterine fluid and within blastocysts. We also evaluate the effect of diet on mTORC1 signalling within blastocysts and examine potential compensatory mechanisms in the blastocyst trophectoderm (TE) lineage. Collectively, our studies indicate a potential role for AA and insulin metabolic signalling through mTORC1 in the induction of blastocyst programming with evidence of increased TE proliferation and invasive behavior in a compensatory response by the time of implantation.

## Results

### Emb-LPD Alters the Composition of Maternal Serum Metabolites

**Figure 1:**
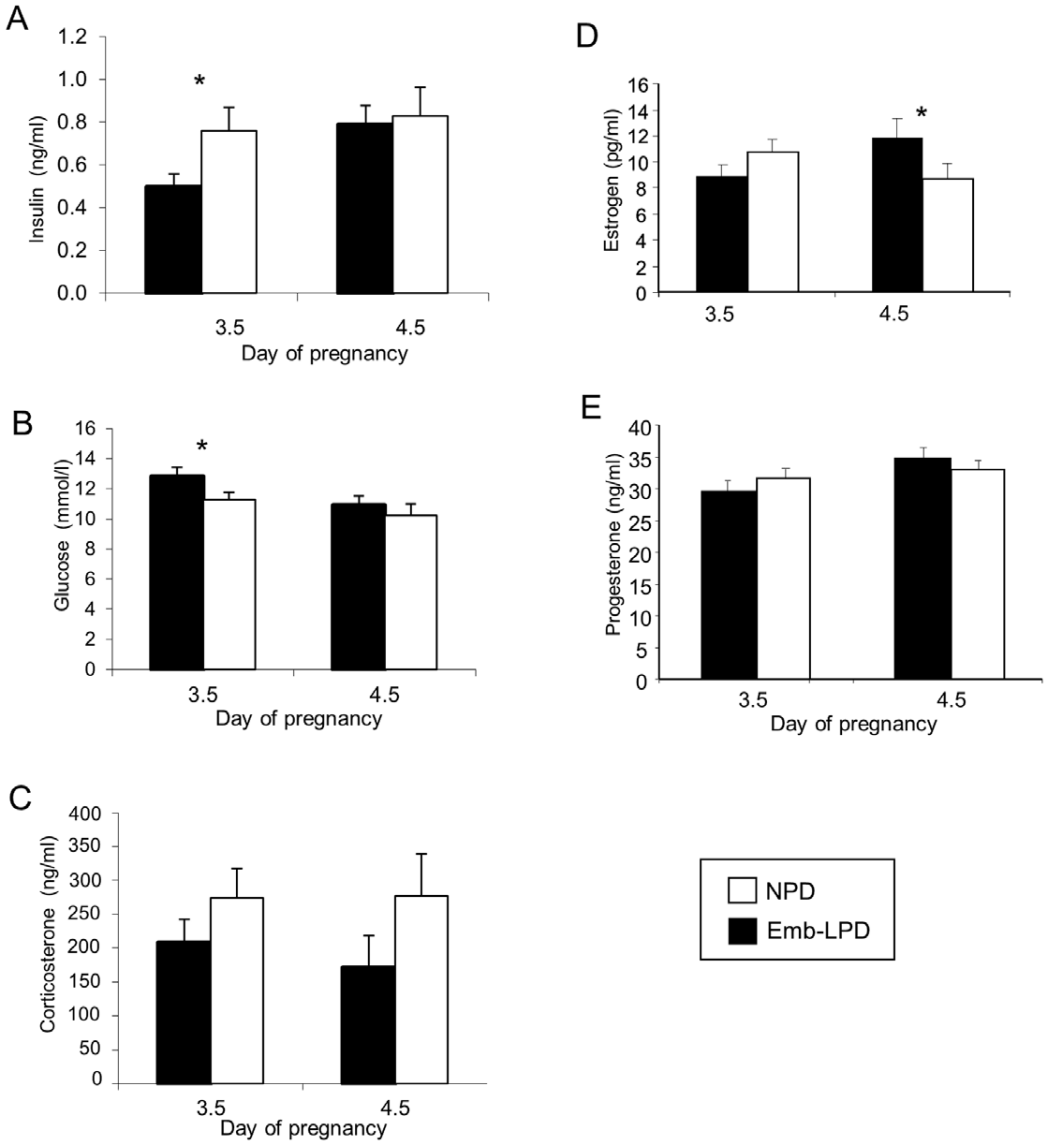
Concentration of maternal serum metabolites at E3.5 and 4.5 following Emb-LPD and NPD treatments. (A) insulin, n = 11–16 per treatment; (B) glucose, n = 13–20 per treatment; (C) corticosterone, n = 11–16 per treatment; (D) estrogen, n = 6–11 per treatment; (E) progesterone, n = 6–11 per treatment. * P<0.05.

Serum metabolites from Emb-LPD and NPD mothers were analysed at E3.5 and 4.5. Emb-LPD serum at E3.5 was depleted in insulin and elevated in glucose (P<0.05; Figure 1A,B). Maternal diet had no effect on corticosterone concentration (Figure 1C); estrogen was increased at E4.5 (P<0.05; Figure 1D) while no change was found in progesterone concentration (Figure 1E).

### Emb-LPD Alters the Composition of Maternal and Blastocyst Amino Acids

**Figure 2:**
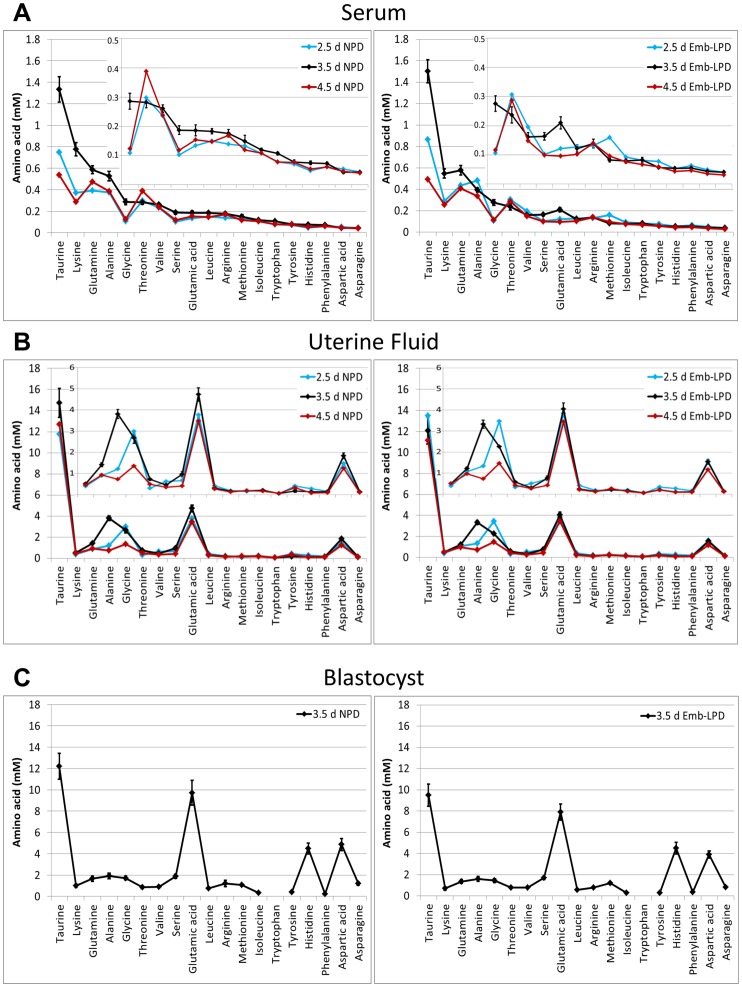
Aminograms in compartments at different time points following Emb-LPD and NPD treatments. (A) Maternal serum, (B) uterine fluid at days 2.5, 3.5 or 4.5 and (C) blastocysts at day 3.5 as taken from Tables 1, 2, 3.

Serum AA composition in Emb-LPD and NPD mothers was analysed at E2.5, 3.5 and 4.5 (Table 1) and further represented as an aminogram with descending AA concentrations at these time points (Figure 2A). No differences in individual AA concentration were evident at E2.5 but at E3.5, alanine, histidine, isoleucine, leucine, lysine, methionine, phenylalanine, tryptophan, tyrosine and valine were all depleted (P<0.05 or lower) in Emb-LPD versus NPD treatment with arginine depleted in Emb-LPD at trend level (P<0.1). At E4.5, isoleucine, leucine, tyrosine and valine were depleted in Emb-LPD versus NPD serum (P<0.05 or lower) while asparagine, phenylalanine and threonine were depleted in Emb-LPD at trend level (P<0.1) (Table 1). At E3.5, combined essential AAs and branched chain AAs were depleted in the Emb-LPD serum (P<0.01 or lower) while at E4.5, branched chain AAs were depleted in Emb-LPD serum (P<0.05) and also essential and total AAs depleted in Emb-LPD serum at trend level (P<0.1). Comparing individual AA concentrations within a treatment group across the time-course showed that several changed as shown in Table 1 (indicated by same letter). The aminogram (Figure 2A) further demonstrates changes in AA concentration across the time course, especially the higher concentration AAs (taurine, lysine, glutamine, alanine, glycine) rising transiently at E3.5.

**Table 1:** Serum concentrations of free amino acids from mice fed either NPD or Emb-LPD at day 2.5, 3.5 or 4.5 of pregnancy.

	Amino acid Concentration (mM)					
	2.5 days		3.5 days		4.5 days	
Amino Acid	NPD	Emb-LPD	NPD	Emb-LPD	NPD	Emb-LPD
n	11	9	14–15^1^	11	10	11
Alanine	0.376±0.062 **a**	0.483±0.061	0.527±0.043 *b	0.395±0.019 *	0.386±0.031	0.338±0.019
Arginine	0.139±0.026	0.132±0.016	0.176±0.013 **▵**	0.139±0.015 **▵**	0.169±0.024	0.140±0.170
Asparagine	0.044±0.009	0.040±0.005	0.043±0.003	0.041±0.003	0.040±0.005 **▵**	0.031±0.003 **▵**
Aspartic acid	0.054±0.007	0.049±0.009	0.043±0.005	0.044±0.006	0.046±0.008	0.035±0.003
Glutamic acid	0.134±0.032	0.122±0.029 **b**	0.186±0.019	0.210±0.021 **a**	0.154±0.027	0.096±0.008 **b**
Glutamine	0.392±0.077 **a**	0.437±0.066	0.588±0.037 **b**	0.578±0.043 **a**	0.473±0.037	0.407±0.029 **a**
Glycine	0.108±0.020 **b**	0.106±0.014 **b**	0.286±0.027 **a**	0.276±0.027 **a**	0.123±0.110 **b**	0.116±0.020 **c**
Histidine	0.048±0.009 **a**	0.053±0.007	0.075±0.006 *b	0.054±0.004 *	0.054±0.005	0.044±0.005
Isoleucine	0.107±0.018	0.093±0.012	0.119±0.006 **‡**	0.079±0.007 **‡**	0.108±0.011 *	0.076±0.010 *
Leucine	0.150±0.024	0.128±0.016	0.183±0.009 **‡**	0.122±0.012 **‡**	0.147±0.015 *	0.102±0.013 *
Lysine	0.370±0.090 **b**	0.292±0.064 **b**	0.777±0.062 **†a**	0.547±0.043 **†a**	0.286±0.068 **b**	0.255±0.063 **b**
Methionine	0.133±0.021	0.160±0.018 **a**	0.149±0.020 **†**	0.083±0.007 **†b**	0.119±0.013	0.096±0.011 **b**
Phenylalanine	0.062±0.009	0.063±0.008	0.073±0.004 **†**	0.055±0.004 †	0.061±0.006 **▵**	0.047±0.005 **▵**
Serine	0.102±0.015 **b**	0.102±0.015 **b**	0.187±0.015 **a**	0.164±0.013 **a**	0.118±0.015 **b**	0.099±0.012 **b**
Taurine	0.749±0.069 **b**	0.863±0.067 **b**	1.333±0.117 **a**	1.501±0.107 **a**	0.538±0.38 **b**	0.493±.020 **b**
Threonine	0.298±0.052	0.306±0.040	0.282±0.018	0.237±0.028	0.388±0.294 **▵**	0.287±0.029 **▵**
Tryptophan	0.079±0.012 **b**	0.082±0.012	0.107±0.005 *a	0.083±0.008 *	0.078±0.006 **b**	0.067±0.008
Tyrosine	0.071±0.013	0.077±0.012	0.078±0.007 *	0.057±0.004 *	0.077±0.008 *	0.059±0.003 *
Valine	0.235±0.037	0.195±0.026	0.261±0.013 **‡**	0.162±0.015 **‡**	0.238±0.024 **†**	0.148±0.015 **†**
Branched chain	0.492±0.048	0.415±0.053	0.563±0.028 **‡**	0.364±0.034 **‡**	0.493±0.05*	0.326±0.048*
Essential	1.434±0.240	1.317±0.182	1.986±0.123 **†a**	1.367±0.104 **†**	1.425±0.126 **▵b**	1.079±0.143 **▵**
Non-essential	2.215±0.332 **b**	2.465±0.266	3.552±0.228 **a**	3.457±0.202 **a**	2.178±0.173 **b**	1.859±0.112 **b**
Total	3.650±0.563 **b**	3.781±0.436	5.538±0.318 **a**	4.842±0.291 **a**	3.604±0.243 **▵b**	2.938±0.242 **▵b**

Values shown with SEM. Differences between diet treatment at individual time points represented by * = P<0.05; † = P<0.01; ‡ = P<0.001; trend ▵ = p<0.1. Differences between time points within the same diet group represented by different letter (P<0.05).

1 3.5 days NPD: n = 14 for Alanine, Threonine, essential, non-essential and total amino acids.

The AA concentration of uterine fluid (UF) was analysed with respect to maternal diet at E2.5, 3.5 and 4.5 (Table 2, Figure 2B). No differences were found between diet treatments for individual AA concentrations at E2.5. At E3.5, the three branched chain AAs, isoleucine, leucine and valine, both individually and collectively, were depleted (P<0.05 or lower) in Emb-LPD versus NPD UF. In addition, glycine, histidine, lysine, taurine and threonine were depleted in Emb-LPD UF at trend level (P<0.1). Both essential and non-essential AAs were depleted in Emb-LPD UF (P<0.05). At E4.5, isoleucine, leucine and valine remained depleted in Emb-LPD versus NPD UF (P<0.05 or lower) while methionine was elevated (P<0.01) in Emb-LPD UF. As was found with maternal serum, changes in concentration of several AAs within treatment group were also evident across the time course (Table 2 Figure 2B). The pattern of transient increase at E3.5 of the higher concentration AAs in serum was not apparent in the UF aminogram except for alanine (Figure 2B). Glycine concentration was higher at E2.5 than E4.5 for both diet treatments (Figure 2B).

**Table 2:** Uterine fluid concentrations of free amino acids from mice fed either NPD or Emb-LPD at 2.5, 3.5 or 4.5 days of pregnancy.

	Amino acid Concentration (mM)					
	2.5 days		3.5 days		4.5 days	
Amino Acid	NPD	Emb-LPD	NPD	Emb-LPD	NPD	Emb-LPD
n	9–10^1^	10	9–12^1^	9–13^1^	10–15^1^	6–15^1^
Alanine	1.208±0.247 **b**	1.333±0.235 **b**	3.803±0.214 **a**	3.321±0.206 **a**	0.726±0.192 **b**	0.736±0.198 **b**
Arginine	0.191±0.021	0.189±0.021 **a**	0.161±0.019	0.142±0.018	0.128±0.017	0.119±0.017 **b**
Asparagine	0.116±0.015	0.120±0.015	0.142±0.014	0.140±0.013	0.105±0.013	0.116±0.013
Aspartic acid	1.476±0.147	1.574±0.147	1.833±0.134 **a**	1.542±0.129	1.253±0.120 **b**	1.172±0.120
Glutamic acid	3.766±0.344	3.834±0.344	4.721±0.314 **a**	4.049±0.301	3.471±0.280 **b**	3.443±0.280
Glutamine	0.894±0.100 **a**	1.074±0.100	1.417±0.091 **b**	1.217±0.088	0.927±0.082 **a**	0.973±0.082
Glycine	2.985±0.302 **a**	3.460±0.287 **a**	2.676±0.262 **▵a**	2.253±0.252 **▵b**	1.348±0.023 **b**	1.468±0.234 **b**
Histidine	0.281±0.028 **a**	0.266±0.028 **a**	0.147±0.025 **▵b**	0.108±0.024 **▵b**	0.089±0.023 **b**	0.092±0.023 **b**
Isoleucine	0.207±0.019	0.196±0.019 **a**	0.212±0.018 *	0.163±0.017 *	0.162±0.016 *	0.133±0.016 *b
Leucine	0.425±0.033 **a**	0.411±0.033 **a**	0.324±0.030 **†**	0.243±0.029 **†b**	0.266±0.027 *b	0.215±0.028 *b
Lysine	0.396±0.052	0.392±0.052	0.521±0.047 **▵**	0.507±0.046 **▵**	0.459±0.042	0.502±0.042
Methionine	0.152±0.025	0.205±0.025	0.184±0.023	0.215±0.022	0.196±0.020 **†**	0.271±0.020 **†**
Phenylalanine	0.169±0.015 **a**	0.167±0.015 **a**	0.139±0.014	0.117±0.014	0.100±0.013 **b**	0.097±0.013 **b**
Serine	0.667±0.135	0.731±0.135	0.966±0.123 **a**	0.764±0.019	0.418±0.110 **b**	0.422±0.110
Taurine	11.757±1.302	13.517±1.302	14.744±1.372 **▵**	12.089±1.372 **▵**	12.709±1.302	11.147±1.681
Threonine	0.305±0.056 **a**	0.326±0.056 **a**	0.726±0.051 **▵b**	0.579±0.049 **▵b**	0.500±0.046 **c**	0.421±0.046
Tryptophan	0.049±0.014	0.046±0.014	0.061±0.013	0.066±0.012	0.074±0.012	0.059±0.012
Tyrosine	0.418±0.173 **a**	0.340±0.073	0.182±0.067 **b**	0.208±0.064	0.332±0.060	0.198±0.060
Valine	0.620±0.044 **a**	0.508±0.044 **a**	0.457±0.040 ‡**b**	0.311±0.039 ‡**b**	0.358±0.036 **†b**	0.269±0.036 **†b**
Branched chain	1.252±0.090 **a**	1.116±0.090 **a**	0.993±0.082 **†**	0.716±0.079 **†b**	0.785±0.073 **b**	0.594±0.076 **b**
Essential	2.321±0.197	2.251±0.197	2.623±0.180 *	2.201±0.173 *	2.114±0.161	1.912±0.161
Non-essential	23.863±2.217	26.438±2.104	31.098±2.217 *a	24.724±2.217 *	21.631±2.104 **b**	19.221±2.716
Total	26.044±2.365	28.689±2.244	33.747±2.365 **a**	26.639±2.365	23.739±2.244 **b**	21.082±2.897

Values shown with SEM. Differences between diet treatment at individual time points represented by * = P<0.05; † = P<0.01; ‡ = P<0.001; trend ▵ = P<0.1. Differences between time points within the same diet group represented by different letter (P<0.05).

1 2.5 days NPD: n = 9 for Alanine, Glycine, non-essential and total amino acids; 3.5 days NPD and Emb-LPD: n = 9 for Taurine, non-essential and total amino acids; 4.5 days NPD and Emb-LPD: n = 10 or 6 for Taurine, non-essential and total amino acids; 4.5 days Emb-LPD: n = 14 for Alanine, Leucine, essential and branched amino acids.

Collected blastocysts at E3.5 were rapidly washed and immediately processed for AA composition (Table 3, Figure 2C). This analysis revealed that the individual AA asparagine was depleted (P<0.05) in concentration within Emb-LPD blastocysts while hypotaurine, lysine and taurine were depleted in Emb-LPD blastocysts at trend level (P<0.1). When individual AA concentrations were converted to relative % of total AAs, lysine and taurine were depleted in Emb-LPD blastocysts (P<0.05), while glutamic acid, phenylalanine (P<0.05), methionine (P<0.001) and valine (P<0.1) were elevated (Table 3). Non-essential AAs were depleted (P<0.05) and essential AAs increased (P<0.05) in relative % total in Emb-LPD blastocysts.

**Table 3:** Blastocyst free amino acid concentration and relative % at day 3.5 of pregnancy from mice fed either NPD or Emb-LPD from morning after mating.

	Amino acid levels at E3.5			
	Concentration (mM)		Relative (%)	
Amino Acid	NPD	Emb-LPD	NPD	Emb-LPD
n	14–16^1^	13–19^1^	14	13
Alanine	1.910±0.264	1.590±0.229	3.690±0.220	3.760±0.370
Arginine	1.210±0.308	0.777±0.0934	2.520±0.390	2.010±0.230
Aspargine	1.200±0.149 *	0.832±0.107 *	2.560±0.170	2.270±0.170
Aspartic acid	4.860±0.551	3.900±0.325	10.800±0.680	10.800±0.270
Glutamic acid	9.71±1.160	7.890±0.759	19.500±0.900 *	22.300±0.710 *
Glutamine	1.660±0.253	1.370±0.178	3.240±0.320	3.330±0.210
Glycine	1.720±0.174	1.450±0.177	3.730±0.310	3.150±0.220
Histidine	4.500±0.506	4.500±0.548	9.090±0.750	9.550±0.460
Hypotaurine	2.070±0.308 **▵**	1.450±0.206 **▵**	4.310±0.420	4.110±0.260
Isoleucine	0.329±0.036	0.295±0.031	0.700±0.030	0.740±0.040
Leucine	0.736±0.086	0.586±0.044	1.510±0.080	1.540±0.130
Lysine	1.000±0.151 **▵**	0.708±0.137 **▵**	2.120±0.230 *	1.450±0.100 *
Methionine	1.080±0.096	1.220±0.123	2.250±0.140 **‡**	3.430±0.220 **‡**
Phenylalanine	0.239±0.024	0.351±0.055	0.550±0.050 *	0.720±0.050 *
Serine	1.870±0.199	1.710±0.171	4.040±0.310	4.250±0.280
Taurine	12.200±1.210 **▵**	9.490±1.050 **▵**	25.100±1.200 *	22.100±0.840 *
Threonine	0.842±0.096	0.789±0.081	1.660±0.140	1.930±0.080
Tyrosine	0.399±0.088	0.311±0.063	0.890±0.300	0.560±0.020
Valine	0.887±0.084	0.785±0.061	1.850±0.040 **▵**	1.980±0.050 **▵**
Branched chain	1.95±0.199	1.670±0.128	4.060±0.120	4.260±0.200
Essential	4.980±0.501	4.840±0.400	10.600±0.230 *	11.800±0.420 *
Non-essential	41.400±4.730	33.500±3.630	89.400±0.230 *	88.200±0.420 *
Total	46.300±5.240	39.400±4.100		

Values shown with SEM. Differences between diet treatment at individual time points represented by * = P<0.05; † = P<0.01; ‡ = P<0.001; ▵ = trend, P<0.1.

1 NPD: n = 15 for Phenylalanine, essential amino acids and n = 14 for Hypotaurine, non-essential and total amino acids; Emb-LPD: n = 18 for Lysine, Methionine, Tyrosine, n = 17 for Alanine, Histidine, Phenylalanine, n = 16 for essential amino acids, n = 15 for Hypotaurine, n = 14 for non-essential amino acids, n = 13 for total amino acids.

**Figure 3:**
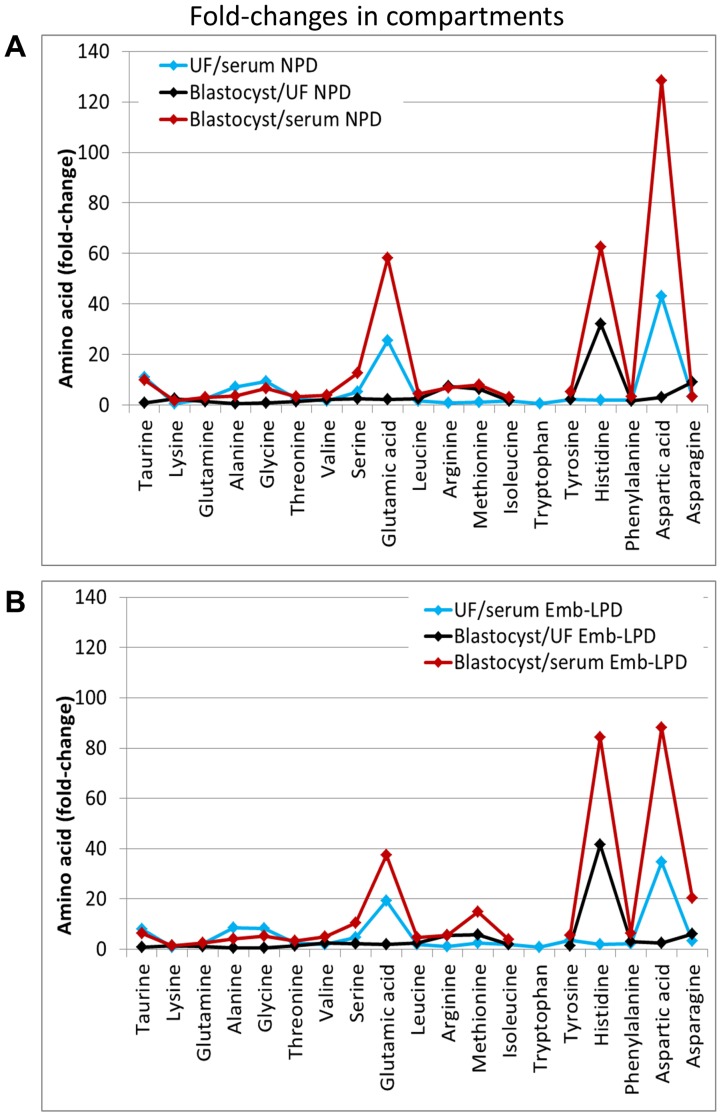
Aminograms of compartments relative to each. (A) Emb-LPD and (B) NPD treatments on day d3.5 as taken from Table 4.

The differing maternal (serum, UF) and blastocyst pools of AAs exhibit marked changes in concentration with blastocysts showing the highest concentration levels for most AAs. In Table 4 and Figure 3, the fold change in concentration for individual and grouped AAs between these pools is recorded for both NPD and Emb-LPD treatments. AA composition of blastocysts and UF was more similar compared to serum.

**Table 4:** Fold changes in concentrations (mM) of free amino acids within serum, uterine fluid and blastocyst compartments at day 3.5 with respect to maternal diet (NPD, Emb-LPD).

	Fold change in amino acid concentration (mM) between compartments					
	UF/serum		blastocyst/UF		blastocyst/serum	
Amino Acid	NPD	Emb-LPD	NPD	Emb-LPD	NPD	Emb-LPD
Alanine	7.217	8.416	0.502	0.479	3.625	4.029
Arginine	0.913	1.025	7.516	5.472	6.860	5.608
Asparagine	3.276	3.438	9.155	5.943	3.276	20.432
Aspartic acid	43.109	34.800	2.979	2.529	128.410	88.014
Glutamic acid	25.432	19.254	2.288	1.949	58.179	37.519
Glutamine	2.410	2.107	1.291	1.126	3.112	2.372
Glycine	9.368	8.176	0.717	0.644	6.722	5.262
Histidine	1.956	2.020	32.041	41.667	62.681	84.146
Isoleucine	1.779	2.058	1.736	1.810	3.087	3.724
Leucine	1.767	1.986	2.457	2.412	4.341	4.789
Lysine	0.671	0.926	2.534	1.397	1.699	1.294
Methionine	1.236	2.600	6.467	5.674	7.991	14.753
Phenylalanine	1.908	2.149	1.719	3.000	3.280	6.446
Serine	5.173	4.660	2.453	2.238	12.691	10.429
Taurine	11.059	8.056	0.902	0.785	9.976	6.324
Threonine	2.575	2.446	1.336	1.363	3.441	3.334
Tryptophan	0.564	0.801				
Tyrosine	2.332	3.624	2.236	1.495	5.215	5.419
Valine	1.752	1.920	2.158	2.524	3.780	4.845
Branch chain	1.748	1.969	2.165	2.332	3.464	4.588
Essential	1.191	1.427	1.899	2.267	2.700	3.767
Non-essential	8.756	7.164	1.502	1.354	11.656	9.708
Total	6.205	5.607	1.374	1.482	8.528	8.309

Values based upon means used in Tables 1, 2 and 3.

### Emb-LPD Induces a Change in mTORC1 Signalling in Blastocysts

**Figure 4:**
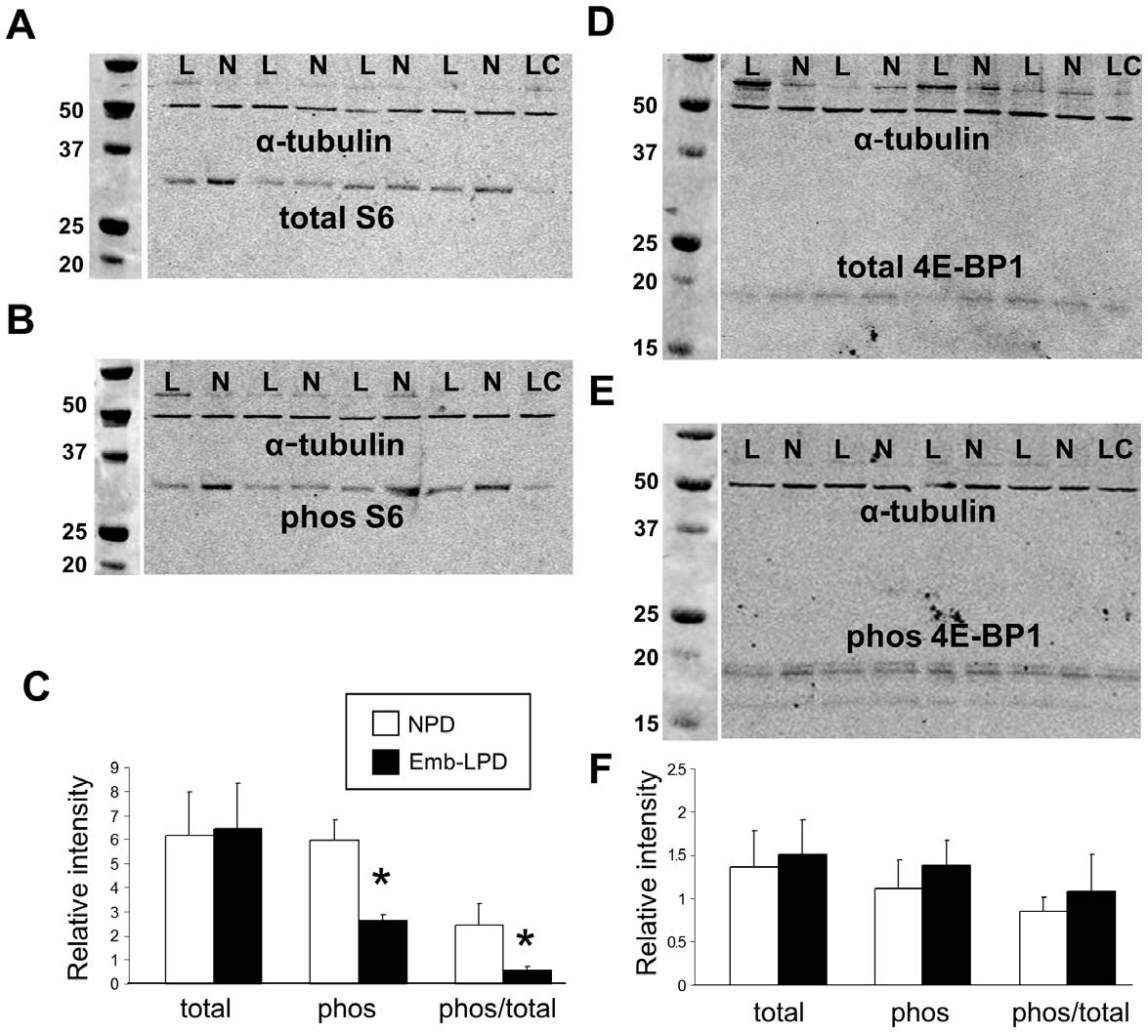
Maternal diet effects on mTORC1 signalling. Quantitative immunoblotting of mTORC1 downstream targets (A–C) S6 protein, (D–F) 4E-BP1 protein normalised to α-tubulin. Representative blots for (A) total S6; (B) phosphorylated S6; (C) relative intensity of total, phosphorylated and ratio of S6 in Emb-LPD and NPD blastocysts; (D) total 4E-BP1; (E) phosphorylated 4E-BP1; (C) relative intensity of total, phosphorylated and ratio of 4E-BP1 in Emb-LPD and NPD blastocysts. Individual blot lanes include MW markers (left) and samples of Emb-LPD (L) and NPD (N) blastocysts (25 blastocysts per lane) and loading control (LC, pooled blastocysts at 25 per lane). * P<0.05, n = 8 samples per treatment.

Collectively, the maternal AA and metabolite studies revealed reduced availability of insulin and branched chain AAs in response to Emb-LPD at E3.5, during the period of blastocyst formation. The mTORC1 pathway regulates protein translation and growth rates within cells and is activated both by insulin and branched chain AAs via PI3K and Akt [43]–[45]. We next investigated whether the depleted nutrient environment following Emb-LPD treatment influenced mTORC1 signalling within blastocysts. We examined the expression and extent of phosphorylation of the mTORC1 downstream targets S6 ribosomal protein (a translation activator, mediated by S6 kinase phosphorylation, of terminal oligopyrimidine (TOP)-dependent transcripts encoding ribosomal proteins commonly involved in cell cycle progression and translation regulation) [46], [47] and 4E-BP1 (a translation repressor protein that inhibits translation by binding to eIF4E translation initiation factor, an interaction abolished by phosphorylation, leading to cap-dependent mRNA translation) [45] using quantitative immunoblotting normalised to α-tubulin. Blastocysts derived from NPD and Emb-LPD mothers exhibited equivalent amounts of total S6 (Figure 4A–C) and 4E-BP1 (Figure 4D–F). However, the pool of phosphorylated S6 within Emb-LPD blastocysts was reduced as was the phosphorylated/total ratio (P<0.05) compared with NPD blastocysts (Figure 4C). In contrast, the extent of phosphorylation of 4E-BP1 was not altered by maternal diet (Figure 4F). These data indicate that the reduced maternal insulin and AA composition in Emb-LPD mothers is associated with reduced mTORC1 signalling capacity by blastocysts.

### Emb-LPD Leads to Change in Blastocyst Phenotype and Outgrowth Potential

**Figure 5:**
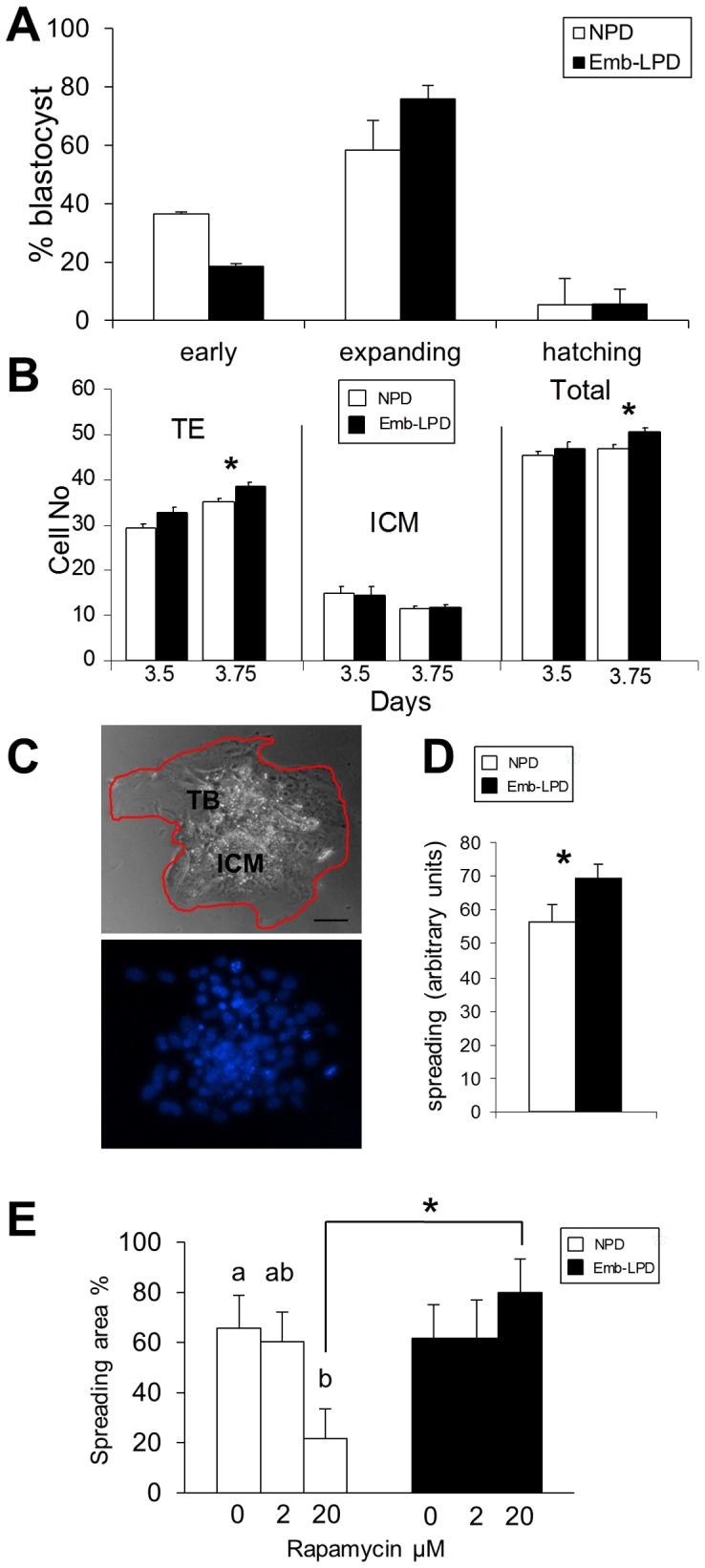
Maternal diet effects on blastocysts and outgrowths. (A) Emb-LPD and NPD blastocyst maturity at E3.5. n = 13–15 mothers per treatment. (B) Emb-LPD and NPD blastocyst cell number at E3.5 and E3.75 within trophectoderm (TE) and ICM and total pools, * P<0.05. n = 135–156 embryos from 11–12 mothers. (C) Representative brightfield image of blastocyst outgrowth showing trophoblast (TB) and ICM cells and with perimeter line included for area measurement, plus corresponding DAPI image for nuclei; Scale bar = 20 µm. (D) Area of outgrowths at 72 h culture, * P<0.05. n = 34–41 per treatment. (E) Area increase during 48–72 h culture, expressed as % change from 48 h, in different concentrations of rapamycin, different letters or * P<0.05. n = 18–23 per treatment.

The number and stage of embryos collected from NPD and Emb-LPD mothers after natural mating at E3.5 over several experiments did not differ significantly (for example, embryos/mouse: NPD 11.9±0.7, Emb-LPD 12.2±0.9; n = 13–15 mothers per treatment). Similarly, the proportion of early, expanding and hatching blastocysts at E3.5 was not significantly different across treatments although the proportion of Emb-LPD blastocysts expanding was slightly higher than NPD blastocysts (Figure 5A). Blastocysts collected at E3.5 and 3.75 were analysed for trophectoderm (TE) and inner cell mass (ICM) cell numbers. TE and ICM cell numbers were equivalent at E3.5 but TE cell numbers and total cell numbers were increased in response to Emb-LPD at E3.75 (P<0.05; Figure 5B). Analysis of late morulae (E3.25) or earlier stage embryos did not show cell number differences between treatments (data not shown).

Blastocysts at E3.5 collected from Emb-LPD and NPD mothers were cultured individually in a modified KSOM+FCS for up to 4 days during which hatching from the zona pellucida, attachment to the culture surface and spreading of trophoblast cells from the TE were monitored. KSOM was modified with the addition of 19 AAs at concentrations equivalent to those found within UF in NPD mothers at E3.5 (KSOMufaa+FCS; see Table 2 for AA composition) for both diet treatment groups. The capacity for embryos to hatch, attach or initiate spreading over the culture surface was not significantly different between treatment groups (for example, % hatched, attached or spreading at 48 h and 72 h culture: NPD 45.9±10.4, 63.8±9.4; Emb-LPD 47.3±9.3, 62.1±8.2; n = 158–179 embryos/treatment). The spreading area of individual outgrowths was measured at 48 h and 72 h culture. This demonstrated no difference between treatments at 48 h but at 72 h, outgrowths derived from Emb-LPD blastocysts had spread over a larger area than those from NPD blastocysts (P<0.05; Figure 5C,D). After 96 h, neither spreading area (NPD 59.9±5.4 and Emb-LPD 59.8±4.5 arbitrary units, n = 26–31) nor number of nuclei (NPD 77.2±4.8 and Emb-LPD 76±4.5 nuclei, n = 27–33) were different between diet treatment and represented when the cultures were no longer expanding in size. To determine whether spreading capacity was regulated via the mTORC1 pathway, blastocyst outgrowths were analysed in the presence or absence of rapamycin, an inhibitor of mTORC1. Rapamycin did not inhibit hatching, attachment or flattening in preparation to spread in either diet group. However, spreading during the 48–72 h period was sensitive to rapamycin (20 µM but not 2 µM) in NPD outgrowths (P<0.05) but was insensitive to rapamycin in the Emb-LPD outgrowths at both concentrations (Figure 5E). Collectively, these data indicate that maternal Emb-LPD induced responses in embryos in vivo including increased TE proliferation and subsequent increased trophoblast spreading capacity, and the latter appeared to be independent of mTORC.

## Discussion

In this study we have investigated the mechanisms by which maternal Emb-LPD may initiate developmental programming within the mouse blastocyst, shown previously to lead to adult disease affecting cardiovascular, metabolic and behavioural systems [15]–[17]. Given that adult disease risk following Emb-LPD is positively correlated with an earlier compensatory response of fetal growth stimulation induced by the blastocyst stage (see Introduction; [15]), our attention has focused on signalling pathways known to affect growth control during development. Our analyses of the effects of the diet on maternal metabolites and in particular AA composition within maternal serum, UF and blastocysts (the first such comparative analysis reported) and associated effects on embryo phenotype and signalling activity, implicate insulin and AA composition as primary upstream factors contributing to developmental programming.

Insulin is known to stimulate mouse preimplantation embryo biosynthesis, proliferation, endocytosis and subsequent fetal growth [37], [41], [42], [48] while hyperglycaemia and maternal diabetes compromise early development [49], [50]. AAs likewise have a beneficial effect on early embryo development, contributing to biosynthesis and proliferation as well as protective mechanisms against osmotic, metal ion and reactive oxygen stresses; essential AAs are particularly supportive of development during morula and blastocyst stages [36], [39], [51]. Emb-LPD caused reduced insulin and increased glucose levels in maternal serum at the time of blastocyst formation (E3.5) together with reduced concentrations of 10 individual AAs and the collective concentration of essential AAs and the branched chain AAs (leucine, isoleucine and valine). We also found estrogen to be elevated by Emb-LPD but only by E4.5 and no changes in progesterone or corticosterone concentration were identified.

The mild maternal hyperglycemia and AA depletion mirrors our data from an earlier study of Emb-LPD treatment in the rat including a consistent serum phenotype of depleted essential and branched chain AAs at the time of blastocyst formation [14]. In the current study, we have been able to include AA analysis of UF in relation to maternal diet, collected during the period of blastocyst formation and expansion and likely to be representative of the most immediate environment change experienced by embryos. Our UF data show broad similarity in relative AA composition to that reported previously for non-pregnant, naturally cycling mice [51] although individual concentrations are some 1.5–4 times higher in the current study, possibly reflecting the difference in strain, pregnancy state and method of UF collection. Comparative analysis between serum and UF composition demonstrates that of the ten AAs depleted in Emb-LPD serum at E3.5 days, only three of these, the branched chain essential AAs, leucine, isoleucine and valine, remain significantly depleted in Emb-LPD UF at this time point. Whilst serum and UF AA concentrations were found not to be different with respect to diet at E2.5, presumably reflecting the short duration of dietary challenge at that time, the data at E4.5 confirmed that obtained at E3.5 with only the three branched chain AAs significantly depleted in UF. Moreover, it is worth emphasising that Emb-LPD induces a global reduction in AA concentration available to embryos in both serum and UF datasets. For example, at E3.5, 16 of the 19 AAs analysed in serum and UF showed mean concentrations reduced following Emb-LPD versus NPD. However, whilst global reductions in AA concentrations are evident at E3.5, and in particular for the branched chain AAs, methionine concentration was elevated in response to Emb-LPD in UF at all three time points and significantly so at E4.5.

The changing patterns of branched chain and methionine AA concentration with respect to diet in serum and UF pools have potential to be contributory to developmental programming mechanisms. Previously, we showed that blastocysts transferred at E3.5 from Emb-LPD mothers to NPD recipients exhibited stimulated growth in late gestation, an early marker for adult disease [15]. This indicates that AA concentrations evident in UF at this time point are more likely to be influential in programming mechanisms. The consistency in branched chain AA concentration depletion across serum and UF during blastocyst formation also previously shown in the rat study [14] together with the concurrent reduction in maternal insulin all implicate mTORC1 complex signalling in blastocyst programming. Branched chain AAs (especially leucine) and insulin activate cellular growth and proliferation to match nutrient availability via mTORC1 serine-threonine kinase which increases the initiation of translation of cap-dependent and terminal oligopyrimidine (TOP)-dependent mRNAs through phosphorylation of the major downstream targets 4E-BP1 and S6 kinase [43]–[45], [47]. AAs may activate mTORC1 via poorly-defined extracellular or intracellular sensor mechanisms [47], [52] while insulin acts directly through the insulin receptor and IRS/PI3K/AKT/mTORC1 pathway [53]. Thus, the combined deficiency in leucine, other branched chain AAs and insulin concentrations in the embryo environment following Emb-LPD may act upstream as negative maternal regulators of nutrient sensing leading to developmental programming. Moreover, mTORC1 signalling mediated through leucine and arginine is activated in blastocysts to control trophoblast motility [38]–[40] and embryos null for the mTOR gene arrest at E5.5 with implantation failure [54].

We evaluated directly the effect of Emb-LPD on blastocyst mTORC1 signalling using antibodies to detect relative pools of phosphorylated and total 4E-BP1 and S6 protein. This analysis demonstrated that blastocysts from Emb-LPD mothers at E3.5 had equivalent amounts and ratio of phosphorylated and total 4E-BP1 to controls from NPD mothers. However, Emb-LPD blastocysts had deficient phosphorylated S6 and its ratio to total S6 protein was reduced compared with NPD controls. Thus, the suppressed levels of insulin and/or AA mTORC1 activators in vivo is clearly sensed by Emb-LPD blastocysts via the S6 effector even after collection and analysis in vitro. Phosphorylated S6 (40S ribosomal protein) acts to stimulate translation of TOP mRNAs which commonly encode translation factors including ribosomal proteins [47]. Whilst both S6 and 4E-BP1 effectors are usually receptive simultaneously in mTORC1 signalling studies, differential responsiveness has been previously reported. Thus, loss of S6 but not 4E-BP1 phosphorylation in response to direct mTORC1 inhibition occurred in urothelial carcinoma lines whereas additional upstream inhibition of PI3K/AKT was required for 4E-BP1 inhibition [55]. Indeed, recent models of mTORC1 effector function suggest distinct biological roles and signalling pathways for the two main effectors S6 and 4E-BP1 [56], [57] as in the current study.

The increase in methionine detected only at 4.5 days in UF may also be pertinent as a contributory factor to onset of developmental programming. LPD, when administered from 2 weeks before mating, has been shown to increase serum homocysteine in mice at the time of blastocyst formation which may in turn lead to folate deficiency and reduced genomic methylation [58]. Homocysteine elevation in this study was proposed to derive from significantly reduced threonine availability to drive its re-methylation to methionine [58]. Reduced serum threonine was detected following LPD in our previous rat study [14] but was only evident at trend level in the current study in serum and UF; however the increase in Emb-LPD UF methionine at E4.5 may be symptomatic of this conversion.

The analysis of blastocyst AA composition (Table 3) revealed a distinct pattern compared with that of maternal serum and UF. This pattern of relative concentration is consistent with those from earlier studies of AA content in mouse blastocysts which reported high concentrations of taurine, glutamic acid and aspartic acid [59], [60]. Here, whilst a global depletion in individual AA concentration in blastocysts was apparent at E3.5 in response to Emb-LPD (17 of 19 AAs analysed) together with reduced pools of essential, non-essential, branched chain and total AA concentrations, these were not significant, reflecting greater variation between samples. A significant reduction in asparagine concentration was, however, detected in Emb-LPD blastocysts. Asparagine was only reduced at trend level in Emb-LPD serum at E4.5 and not affected by diet in UF. Further, hypotaurine, lysine and taurine were reduced in Emb-LPD blastocysts at trend level compared with NPD. These AAs have been shown either to be consumed by blastocysts or to be supportive of their development [61]–[66] and so may also contribute to diet-induced altered blastocyst phenotype and potential.

The blastocyst AA composition indicated that despite the reduced maternal availability of branched chain AAs by Emb-LPD and the accompanying reduction in mTORC1 signalling, these AAs were nonetheless present within blastocysts at near normal concentrations. This distinction indicates the importance of these AAs to blastocyst metabolism and presumably reflects an altered blastocyst uptake rate or transporter expression profile to compensate. It is thought nutrient sensing via mTORC1 may be mediated at cell surface or intracellular sites [47], [52] thereby allowing both mTORC1 signalling and compensatory activity to occur concurrently.

Comparative analysis of the serum, UF and blastocyst AA concentrations within a single study has not been reported previously and demonstrates the dynamic and regulated nature of these pools with significant fold changes occurring between them (Table 4; Figure 4). Total and most individual AAs increase in concentration from serum to UF (∼6 fold) and from UF to blastocyst (∼1.4 fold), a trend previously reported [51], [59] and reflecting the regulatory epithelial transport boundaries provided by endometrium and trophectoderm [39], [67], [68]. Whilst the biological contribution of these changing pools to reproductive function has been reviewed previously, it is worth emphasising the importance of AA composition in culture media reflecting in vivo conditions close to the embryo [51]. We are currently utilising the UF AA composition (Table 3) in in vitro studies on developmental potential.

A final component of our AA analysis was to construct aminograms of the AA concentration in the three compartments (serum, UF, blastocysts) with respect to diet (Figure 2). These facilitate comparison of pattern of AA concentration change across the compartments and with respect to time points used. Notably, the serum aminogram revealed a transient peak in the more abundant AAs (taurine, lysine, glutamine, alanine, glycine) at E3.5 relative to E2.5 and E4.5 (Figure 2A). However, this pattern was generally not maintained within UF except for alanine and partially for glycine (occurring at ∼7-fold and ∼9-fold higher concentration in UF relative to serum, respectively). Alanine and glycine are both supportive of embryo development and facilitate osmoregulation to provide cell volume control in late cleavage [69] and their high local concentration may become critical to maintain cell volume around the time of blastocyst formation when fluid transport into the embryo is active. The compartment aminograms also show multi-fold increased levels of glutamic acid and aspartic acid in UF and blastocysts relative to serum and histidine in blastocysts relative to serum and UF. Glutamic and aspartic acids are recognised abundant constituents of maternal tract fluid [51] and their concentration in blastocysts will likely reflect this. Histidine, through conversion to histamine, indirectly contributes to blastocyst-uterine signalling at implantation [70] and is further implicated in blastocyst growth [71].

Our analysis of blastocyst and outgrowth phenotype in response to Emb-LPD revealed that TE and total cell numbers increased at 3.75 days and that outgrowth spreading increased over 48–72 hours following culture of blastocysts (Figure 5). The increase in blastocyst cell numbers did not include those within the ICM which were slightly lower at E3.75 than at E3.5 in both treatment groups. ICM cell number in this strain of outbred mice (MF1) does not increase synchronously with time during blastocyst expansion reflecting the cell cycle duration (∼12 hr) and relative position of individual cells within that cycle [72]; this may explain the absence of proliferation in the ICM over the two time points used. The preferential increased proliferation of the extra-embryonic TE cells and their subsequent increased motility may be viewed as an early compensatory response to poor diet to enhance early trophoblast invasive capacity at implantation. Indeed, evidence of compensatory placental morphological organisation and transport activity in response to LPD in later gestation was reported recently [73]. This early response would augment the compensatory activity we identified previously in endocytotic activity and receptor expression of the extra-embryonic visceral endoderm lineage in late gestation in response to Emb-LPD and LPD. Our blastocyst cell number analysis is also broadly consistent with an earlier mouse study where maternal dietary protein level was reduced from 3–4 weeks before mating resulting in a reduced blastocyst ICM/TE ratio [74]. However, in our rat model, both ICM and TE cell numbers were reduced by Emb-LPD which may indicate a species difference in TE response coinciding with compensatory growth occurring later than in the mouse, mainly postnatally [14]. The spreading activity was found to be rapamycin-insensitive in Emb-LPD outgrowths while rapamycin was effective in inhibiting spreading in control samples but only at the higher dose used (20 µM). The mTORC1 in mouse blastocysts, shown to be responsive to leucine and arginine to induce rapamycin-sensitive outgrowth activity, has some unusual characteristics in that the complex subunit, LST8, is down-regulated just at the time of kinase activation in the blastocyst and the downstream effectors 4E-BP1 and S6K phosphorylation patterns do not always correlate to outgrowth competency [40]. Moreover, the relatively high concentration of rapamycin, an allosteric inhibitor of mTORC1, required in the current study to demonstrate the diet-related difference in spreading activity is unusual although the threshold of rapamycin sensitivity is variable in different cellular models and evidence of mTORC1 resistance can be explained by reduced capacity to induce or maintain 4E-BP1 dephosphorylation [75]. This may indicate that enhanced activity in Emb-LPD outgrowths may be mediated through the 4E-BP1 pathway, through undefined interactions of the unusual blastocyst mTORC1 or 2 complex, through compensatory feedback loops involving insulin, AKT and mTORC1 and 2 signaling or through loss of rapamycin-sensitivity of one or both mTORC complexes after prolonged exposure. Alternatively, the induction of enhanced trophoblast migration by Emb-LPD may occur either via other related signalling components such as the energy sensing AMPK [76] or earlier, prior to blastocyst collection, since UF from the time of blastocyst expansion is competent to induce subsequent outgrowth activity in medium lacking AAs [40].

In conclusion, we have investigated mechanisms of early induction of adverse long-term developmental programming mediated through Emb-LPD. We provide evidence that dietary-mediated changes in maternal metabolites and AAs, including a reduction in branched chain AA availability in the immediate environment experienced by blastocysts during their formation, may act as a nutrient sensing mechanism for the embryos. We show that this altered environment associates with a reduced mTORC1 signalling capacity and AA content within Emb-LPD blastocysts. Lastly, we show that changes in blastocyst phenotype, notably increased TE proliferation and outgrowth invasiveness behaviour occur in response to Emb-LPD that can be interpreted as early compensatory mechanisms. The stimulation in fetal and postnatal growth as an early marker of adult disease mediated through Emb-LPD and shown to be induced by the blastocyst stage [15] likely reflects first accurate sensing of environmental nutrients by embryos and second activation of compensatory mechanisms. Whilst we interpret altered mTORC1 signalling as an appropriate sensing mechanism and altered TE phenotype as an early compensatory mechanism, we envisage concurrent modulation in embryo metabolism that will convert the negative mTORC1 signal into a positive biosynthetic response to drive compensatory growth. The nature of such modulation in embryo metabolism is currently being investigated.

## Materials and Methods

### Animal Treatments

All mice and experimental procedures were conducted using protocols approved by, and in accordance with, the UK Home Office Animal (Scientific Procedures) Act 1986 and local ethics committee at the University of Southampton under UK Home Office Project Licence PPL30/2467. MF1 mice, under UK Home Office licence and local ethics approval, were bred in-house (University of Southampton) on a 0700–1900 light cycle with standard chow. Virgin females (7–8.5 weeks) were mated naturally overnight with MF1 males and plug positive females housed individually the following morning and assigned without preference to either (a) normal protein diet (NPD, 18% casein) or (b) low protein diet (Emb-LPD, 9% casein) (Special Diet Services, Lillico Biotechnology, UK) for up to E4.5. Diets were isocaloric and their full composition has been described previously [15], [77].

### Metabolic Assays

Serum was derived from maternal blood after culling and heart puncture at E3.5 and 4.5 in both diet treatment groups and stored at −80°C before use in metabolic assays. Glucose concentrations were determined using the glucose oxidase GOD-Perid method with a Cobas-Mira S autoanalyser (Roche Instruments, UK). Insulin was measured using the rat insulin sandwich ELISA kit (Crystal Chem International, IL, USA) and a Dynatech MR5000 plate reader. Corticosterone was measured using an ^125^I RIA kit (ICN Biomedicals, UK) and a 1274 RiaGamma counter (LKB-Wallac, Finland). Estrogen was determined using the 3^rd^ Generational Estradiol RIA kit (DSL, UK) and progesterone using the 17α-OH progesterone ^125^I RIA kit (DSL, UK), both counted using a 1274 RiaGamma counter.

### Collection of Uterine Luminal Fluid

Following Emb-LPD or NPD treatment for up to E4.5, females were culled by cervical dislocation and uterine fluid (UF) was collected by direct sampling. Each uterine horn was ligated at the posterior end close to the cervix during dissection before cutting away from the oviduct. The free uterine ends were washed in ice-cold phosphate buffered saline (PBS) and blotted before removing one ligature and gently inserting a collection pipette (see below) into the lumen down to the cervical junction and slowly removing it with UF collected by capillary action. This was repeated for the other horn. The collection pipette was constructed from a flame-pulled region (∼0.5 mm outer diameter) of a Pasteur pipette, subsequently flame-polished at the tip and calibrated using diluted [^14^C]-sucrose to relate internal pipette fluid volume determined by microscopy using an eyepiece graticule with radioactivity using an LS6500 Gamma counter (Beckman, UK). The volume of collected UF was recorded before diluting in PBS up to 13 µl, centrifuging at 10,000 **g** for 10 min at 4°C to remove any cellular contents, and supernatant (10 µl) and pellet (3 µl) samples stored at −80°C. UF pellet samples were examined for cellular content by immunocytochemistry using epithelial and macrophage antibodies; cellular detection was minimal, predominantly epithelial cells, approximating 5 cells/sample with no relationship evident with maternal diet treatment (data not shown).

### Embryo Collection and Treatments

Embryos were collected at different times during dietary treatment from mothers by cervical dislocation, dissection of reproductive tract and flushing of embryos using H6 medium supplemented with 4 mg/ml BSA (H6-BSA) [9]. For analysis of free amino acid composition, blastocyst stage and diameter were assessed (early, mid, expanded) followed by three thorough washes in PBS supplemented with 0.1% PVA before flash-freezing in groups of 8–10 (separated by per mother) in 10 µl of HPLC-grade water in HPLC-vials on dry ice as rapidly as possible. This procedure took less than 10 min per mother and wash drop analysis showed no or background traces of AA even if embryos were left for up to 30 min suggesting no or negligible leakage during the collection process.

For assessing cell numbers, embryos were subjected to differential nuclear labeling after complement-mediated lysis of the trophectoderm [78] with modifications. Briefly, embryos were placed in 10% tetranitrobenzene sulphonic acid (TNBS; Sigma, UK) in H6+0.1% PVA for 10 mins, washed 3 times in H6-BSA, incubated in 0.4 mg/ml rabbit anti-dinitrophenyl (anti-DNP) antibody (Sigma, UK) in H6-BSA for 10 mins, washed in H6-BSA and incubated in a 25 µl drop of reconstituted Low-Tox guinea pig complement (1∶10 dilution, Cedarlane, Canada) with 2 µl propidium iodide (PI; Sigma, 1 mg/ml) for 10 mins at 37°C. Embryos were washed in H6-BSA, fixed in ethanol plus 1% bisbenzimide (Sigma) at 4°C, washed in fresh ethanol, mounted in glycerol on a glass slide, viewed on a Zeiss Axiophot upright epifluorescent microscope and nuclei counted using Metamoph software (Version 6.2r6).

To evaluate further development and outgrowth potential, blastocysts were placed individually into 20 µl drops of KSOMufaa supplemented with 10% FCS (KSOMufaa+FCS) under oil and allowed to attach and spread for up to 120 h. KSOMufaa comprises KSOM medium (Sigma) supplemented with the mean composition of AAs identified in the UF of NPD mothers at E3.5 (Table 2). Morphology and attachment of blastocysts were assessed at daily intervals using a Zeiss Axiovert inverted microscope fitted with environment chamber at 37°C, and brightfield images taken every 24 h for area measurements using Metamorph software. Blastocyst outgrowth was also conducted for specific periods with medium containing rapamycin (2 or 20 µM; LC laboratories, Woburn, USA) or vehicle (0.05% DMSO) to inhibit mTORC1 activity.

### Amino Acid Analysis

Serum and UF concentrations of 19 AAs were determined using a Kontron 500 series automated reverse phase high performance liquid chromatography system equipped with a Jasco F920 fluorescence detector and a 4.5 mm×250 mm Hypersil ODS-16 column (Jones Chromatography, Glamorgan, UK). Serum and UF samples were diluted from 1/12.5 to 1/112.5 with PBS or HPLC grade water (Fisher Scientific, UK) dependent upon sample type and AAs to be detected; derivatized (precolumn) with *o*-phthaldialdehyde [51], D-homoserine and α-amino butyric acid; run in duplicates and compared with 10 µmol l^−1^ AA standards. For blastocyst AA analysis, a 4.6 mm×250 mm Hyperclone 5u ODS (C18) 120A column (Phenomenex, Macclesfield, UK) was employed. Samples were diluted 1∶1 with HPLC grade water and compared to 10 µM standards and negative controls (washing medium and HPLC water). For calculating absolute concentrations, blastocyst volume was taken into account for each sample.

### Blastocyst mTORC1 Activity

Blastocysts were collected from diet-treated mothers at E3.5 and transferred to SDS sample buffer containing protease (Roche cOmplete mini protease inhibitor cocktail, UK) and phosphatase (Sigma Phosphatase Inhibitor Cocktails 1 and 2, Poole, UK) inhibitors, boiled and stored at −80°C in 25 embryos/15 µl aliquots for each diet treatment (∼10 blastocysts collected per mother following natural mating). Samples were re-boiled and run on 4–12% bis-tris gels (Invitrogen, UK), transferred onto nitrocellulose membrane overnight, blocked in 5% milk in TBS-Tween (0.1% Tween 20) for 1 h before incubation in primary antibodies overnight at 4°C. All primary antibodies were from Cell Signaling (Invitrogen, UK), made up in 5% BSA in TBS-Tween (total S6 ribosomal protein(5G10) #2217 1∶2000; phospho-S6 ribosomal protein(Ser235/236) #4858 1∶2000; total 4E-BP1 #9452, 1∶500–1000; phospho-4E-BP1(Thr37/46) #9459 1∶500–1000; α-tubulin #2144 1∶10,000) with 25 blastocysts equivalent per lane, split from a pool of 50, for total protein and phosphorylated protein blotting respectively of S6 and 4E-BP1 with α-tubulin used as control protein. Inter-gel variation was normalized by running a standard pooled blastocyst sample (25 equivalent per lane) on all gels. Blots were washed in TBS-Tween and incubated in appropriate IRDye-conjugated secondary antibodies (Molecular Probes, Invitrogen, UK), washed and membranes analysed by densitometry using an Odyssey Infrared Imaging System (Licor).

### Statistics

Amino acid composition, embryo cell numbers and outgrowth spreading were analysed using one-way ANOVA or two-way ANOVA (day or drug and diet treatment as factors) followed by appropriate post hoc tests (Bonferroni or Mann-Whitney Rank sum test if data were not normally distributed; SigmaPlot software package version 12.1 and 12.2). All western blotting data were assessed for normality and analysed by t-test (SPSS version 16). Significance was assumed at P<0.05.

## References

[1] Fleming TP, Kwong WY, Porter R, Ursell E, Fesenko I, et al (2004) The embryo and its future. Biol Reprod 71: 1046–54. 15215194 10.1095/biolreprod.104.030957

[2] Sinclair KD, Singh R (2007) Modelling the developmental origins of health and disease in the early embryo. Theriogenology 67: 43–53. 17049592 10.1016/j.theriogenology.2006.09.017

[3] Watkins AJ, Papenbrock T, Fleming TP (2008) The preimplantation embryo: handle with care. Semin Reprod Med 26: 175–185. 18302109 10.1055/s-2008-1042956

[4] Fleming TP, Velazquez MA, Eckert JJ, Lucas ES, Watkins AJ (2012) Nutrition of females during the peri-conceptional period and effects on foetal programming and health of offspring. Anim Reprod Sci 130: 193–197. 22341375 10.1016/j.anireprosci.2012.01.015

[5] Duranthon V, Watson AJ, Lonergan P (2008) Preimplantation embryo programming: transcription, epigenetics, and culture environment. Reproduction 135: 141–150. 18239045 10.1530/REP-07-0324

[6] Ecker DJ, Stein P, Xu Z, Williams CJ, Kopf GS, et al (2004) Long-term effects of culture of preimplantation mouse embryos on behavior. Proc Natl Acad Sci USA 101: 1595–600. 14747652 10.1073/pnas.0306846101 PMC341785

[7] Fernandez-Gonzalez R, Moreira P, Bilbao A, Jiménez A, Pérez-Crespo M, et al (2004) Long-term effect of in vitro culture of mouse embryos with serum on mRNA expression of imprinting genes, development, and behavior. Proc Natl Acad Sci USA 101: 5880–5. 15079084 10.1073/pnas.0308560101 PMC395892

[8] Mahsoudi B, Li A, O'Neill C (2007) Assessment of the long-term and transgenerational consequences of perturbing preimplantation embryo development in mice. Biol Reprod 77: 889–896. 17699738 10.1095/biolreprod.106.057885

[9] Watkins AJ, Platt D, Papenbrock T, Wilkins A, Eckert JJ, et al (2007) Mouse embryo culture induces changes in postnatal phenotype including raised systolic blood pressure. Proc Natl Acad Sci U S A 104: 5449–5454. 17372207 10.1073/pnas.0610317104 PMC1838459

[10] Morgan HD, Jin XL, Li A, Whitelaw E, O'Neill C (2008) The culture of zygotes to the blastocyst stage changes the postnatal expression of an epigentically labile allele, agouti viable yellow, in mice. Biol Reprod 79: 618–623. 18562706 10.1095/biolreprod.108.068213

[11] Fernandez-Gonzalez R, Ramirez MA, Bilbao A, De Fonseca FR, Gutierrez-Adan A (2007) Suboptimal in vitro culture conditions: an epigenetic origin of long-term health effects. Mol Reprod Dev 74: 1149–1156. 17474101 10.1002/mrd.20746

[12] Sjoblom C, Roberts CT, Wikland M, Robertson SA (2005) Granulocyte-macrophage colony-stimulating factor alleviates adverse consequences of embryo culture on fetal growth trajectory and placental morphogenesis. Endocrinology 146: 2142–53. 15705781 10.1210/en.2004-1260

[13] Banrezes B, Sainte-Beuve T, Canon E, Schultz RM, Cancela J, et al (2011) Adult body weight is programmed by a redox-regulated and energy-dependent process during the pronuclear stage in mouse. PLoS One 6: e29388. 22216268 10.1371/journal.pone.0029388 PMC3247262

[14] Kwong WY, Wild AE, Roberts P, Willis AC, Fleming TP (2000) Maternal undernutrition during the preimplantation period of rat development causes blastocyst abnormalities and programming of postnatal hypertension. Development 127: 4195–4202. 10976051 10.1242/dev.127.19.4195

[15] Watkins AJ, Ursell E, Panton R, Papenbrock T, Hollis L, et al (2008) Adaptive responses by mouse early embryos to maternal diet protect fetal growth but predispose to adult onset disease. Biol Reprod 78: 299–306. 17989357 10.1095/biolreprod.107.064220

[16] Watkins AJ, Lucas ES, Torrens C, Cleal JK, Green L, et al (2010) Maternal low-protein diet during mouse pre-implantation development induces vascular dysfunction and altered renin-angiotensin-system homeostasis in the offspring. Br J Nutr 103: 1762–1770. 20128937 10.1017/S0007114509993783

[17] Watkins AJ, Lucas ES, Wilkins A, Cagampang FR, Fleming TP (2011) Maternal periconceptional and gestational low protein diet affects mouse offspring growth, cardiovascular and adipose phenotype at 1 year of age. PLoS One 6: e28745. 22194901 10.1371/journal.pone.0028745 PMC3240629

[18] Gardner DS, Pearce S, Dandrea J, Walker R, Ramsay MM, et al (2004) Peri-implantation undernutrition programs blunted angiotensin II evoked baroreflex responses in young adult sheep. Hypertension 43: 1290–1296. 15078864 10.1161/01.HYP.0000126991.67203.7b PMC2655056

[19] Sinclair KD, Allegrucci C, Singh R, Gardner DS, Sebastian S, et al (2007) DNA methylation, insulin resistance, and blood pressure in offspring determined by maternal periconceptional B vitamin and methionine status. Proc Natl Acad Sci U S A 104: 19351–19356. 18042717 10.1073/pnas.0707258104 PMC2148293

[20] Torrens C, Snelling TH, Chau R, Shanmuganathan M, Cleal JK, et al (2009) Effects of pre- and periconceptional undernutrition on arterial function in adult female sheep are vascular bed dependent. Exp Physiol 94: 1024–1033. 19561141 10.1113/expphysiol.2009.047340

[21] Hernandez CE, Matthews LR, Oliver MH, Bloomfield FH, Harding JE (2010) Effects of sex, litter size and periconceptional ewe nutrition on offspring behavioural and physiological response to isolation. Physiol Behav 101: 588–594. 20826171 10.1016/j.physbeh.2010.08.020

[22] Barker DJ, Osmond C, Golding J, Kuh D, Wadsworth ME (1989) Growth in utero, blood pressure in childhood and adult life, and mortality from cardiovascular disease. BMJ 298: 564–567. 2495113 10.1136/bmj.298.6673.564 PMC1835925

[23] Langley-Evans SC, McMullen S (2010) Developmental origins of adult disease. Med Princ Pract 19: 87–98. 20134170 10.1159/000273066

[24] Godfrey KM, Gluckman PD, Hanson MA (2010) Developmental origins of metabolic disease: life course and intergenerational perspectives. Trends Endocrinol Metab 21: 199–205. 20080045 10.1016/j.tem.2009.12.008

[25] Barker DJ (2007) The origins of the developmental origins theory. J Intern Med 261: 412–417. 17444880 10.1111/j.1365-2796.2007.01809.x

[26] Thompson JG, Mitchell M, Kind KL (2007) Embryo culture and long-term consequences. Reprod Fertil Dev 19: 43–52. 17389134 10.1071/rd06129

[27] Basatemur E, Sutcliffe A (2008) Follow-up of children born after ART. Placenta 29 Suppl B: 135–140. 10.1016/j.placenta.2008.08.013 18790325

[28] Ceelen M, van Weissenbruch MM, Vermeiden JP, van Leeuwen FE, Delemarre-van de Waal HA (2008) Cardiometabolic differences in children born after in vitro fertilization: follow-up study. J Clin Endocrinol Metab 93: 1682–1688. 18285409 10.1210/jc.2007-2432

[29] Dumoulin JC, Land JA, Van Montfoort AP, Nelissen EC, Coonen E, et al (2010) Effect of in vitro culture of human embryos on birthweight of newborns. Hum Reprod 25: 605–612. 20085915 10.1093/humrep/dep456

[30] Hardy K, Spanos S (2002) Growth factor expression and function in the human and mouse preimplantation embryo. J Endocrinol 172: 221–236. 11834440 10.1677/joe.0.1720221

[31] Armant DR (2005) Blastocysts don't go it alone. Extrinsic signals fine-tune the intrinsic developmental program of trophoblast cells. Dev Biol 280: 260–280. 15882572 10.1016/j.ydbio.2005.02.009 PMC2715296

[32] Robertson SA (2007) GM-CSF regulation of embryo development and pregnancy. Cytokine Growth Factor Rev 18: 287–298. 17512774 10.1016/j.cytogfr.2007.04.008

[33] Koga K, Mor G (2008) Expression and function of toll-like receptors at the maternal-fetal interface. Reprod Sci 15: 231–242. 18421019 10.1177/1933719108316391

[34] Guzeloglu-Kayisli O, Kayisli UA, Taylor HS (2009) The role of growth factors and cytokines during implantation: endocrine and paracrine interactions. Semin Reprod Med 27: 62–79. 19197806 10.1055/s-0028-1108011 PMC3107839

[35] Douglas AJ (2011) Mother-offspring dialogue in early pregnancy: impact of adverse environment on pregnancy maintenance and neurobiology. Prog Neuropsychopharmacol Biol Psychiatry 35: 1167–1177. 20688125 10.1016/j.pnpbp.2010.07.024

[36] Lane M, Gardner DK (1997) Differential regulation of mouse embryo development and viability by amino acids. J Reprod Fertil 109: 153–164. 9068427 10.1530/jrf.0.1090153

[37] Kaye PL, Gardner HG (1999) Preimplantation access to maternal insulin and albumin increases fetal growth rate in mice. Hum Reprod 14: 3052–3059. 10601096 10.1093/humrep/14.12.3052

[38] Martin PM, Sutherland AE (2001) Exogenous amino acids regulate trophectoderm differentiation in the mouse blastocyst through an mTOR-dependent pathway. Dev Biol 240: 182–193. 11784055 10.1006/dbio.2001.0461

[39] Martin PM, Sutherland AE, Van Winkle LJ (2003) Amino acid transport regulates blastocyst implantation. Biol Reprod 69: 1101–1108. 12801981 10.1095/biolreprod.103.018010

[40] Gonzalez IM, Martin PM, Burdsal C, Sloan JL, Mager S, et al (2012) Leucine and arginine regulate trophoblast motility through mTOR-dependent and independent pathways in the preimplantation mouse embryo. Dev Biol 361: 286–300. 22056783 10.1016/j.ydbio.2011.10.021 PMC3246567

[41] Kaye PL, Harvey MB (1995) The role of growth factors in preimplantation development. Prog Growth Factor Res 6: 1–24. 8714366 10.1016/0955-2235(95)00001-1

[42] Heyner S (1997) Growth factors in preimplantation development: role of insulin and insulin-like growth factors. Early Pregnancy 3: 153–163. 10086065

[43] Proud CG (2007) Amino acids and mTOR signalling in anabolic function. Biochem Soc Trans 35: 1187–1190. 17956308 10.1042/BST0351187

[44] Wang X, Proud CG (2009) Nutrient control of TORC1, a cell-cycle regulator. Trends Cell Biol 19: 260–267. 19419870 10.1016/j.tcb.2009.03.005

[45] Dowling RJ, Topisirovic I, Fonseca BD, Sonenberg N (2010) Dissecting the role of mTOR: lessons from mTOR inhibitors. Biochim Biophys Acta 1804: 433–439. 20005306 10.1016/j.bbapap.2009.12.001

[46] Peterson RT, Schreiber SL (1998) Translation control: connecting mitogens and the ribosome. Curr Biol 8: R248–250. 9545190 10.1016/s0960-9822(98)70152-6

[47] Kim E (2009) Mechanisms of amino acid sensing in mTOR signaling pathway. Nutr Res Pract 3: 64–71. 20016704 10.4162/nrp.2009.3.1.64 PMC2788159

[48] Dunglison GF, Jane SD, McCaul TF, Chad JE, Fleming TP, et al (1995) Stimulation of endocytosis in mouse blastocysts by insulin: a quantitative morphological analysis. J Reprod Fertil 105: 115–123. 7490702 10.1530/jrf.0.1050115

[49] Pampfer S (2000) Peri-implantation embryopathy induced by maternal diabetes. J Reprod Fertil Suppl 55: 129–139. 10889842

[50] Jungheim ES, Moley KH (2008) The impact of type 1 and type 2 diabetes mellitus on the oocyte and the preimplantation embryo. Semin Reprod Med 26: 186–195. 18302110 10.1055/s-2008-1042957

[51] Harris SE, Gopichandran N, Picton HM, Leese HJ, Orsi NM (2005) Nutrient concentrations in murine follicular fluid and the female reproductive tract. Theriogenology 64: 992–1006. 16054501 10.1016/j.theriogenology.2005.01.004

[52] Taylor PM (2009) Amino acid transporters: eminences grises of nutrient signalling mechanisms? Biochem Soc Trans 37: 237–241. 19143639 10.1042/BST0370237

[53] Cheng Z, Tseng Y, White MF (2010) Insulin signaling meets mitochondria in metabolism. Trends Endocrinol Metab 21: 589–598. 20638297 10.1016/j.tem.2010.06.005 PMC3994704

[54] Gangloff YG, Mueller M, Dann SG, Svoboda P, Sticker M, et al (2004) Disruption of the mouse mTOR gene leads to early postimplantation lethality and prohibits embryonic stem cell development. Mol Cell Biol 24: 9508–9516. 15485918 10.1128/MCB.24.21.9508-9516.2004 PMC522282

[55] Nawroth R, Stellwagen F, Schulz WA, Stoehr R, Hartmann A, et al (2011) S6K1 and 4E-BP1 are independent regulated and control cellular growth in bladder cancer. PLoS One 6: e27509. 22110663 10.1371/journal.pone.0027509 PMC3216974

[56] Duvel K, Yecies JL, Menon S, Raman P, Lipovsky AI, et al (2010) Activation of a metabolic gene regulatory network downstream of mTOR complex 1. Mol Cell 39: 171–183. 20670887 10.1016/j.molcel.2010.06.022 PMC2946786

[57] Sengupta S, Peterson TR, Sabatini DM (2010) Regulation of the mTOR complex 1 pathway by nutrients, growth factors, and stress. Mol Cell 40: 310–322. 20965424 10.1016/j.molcel.2010.09.026 PMC2993060

[58] Petrie L, Duthie SJ, Rees WD, McConnell JM (2002) Serum concentrations of homocysteine are elevated during early pregnancy in rodent models of fetal programming. Br J Nutr 88: 471–477. 12425727 10.1079/BJN2002695

[59] Van Winkle LJ, Dickinson HR (1995) Differences in amino acid content of preimplantation mouse embryos that develop in vitro versus in vivo: in vitro effects of five amino acids that are abundant in oviductal secretions. Biol Reprod 52: 96–104. 7711189 10.1095/biolreprod52.1.96

[60] Schultz GA, Kaye PL, McKay DJ, Johnson MH (1981) Endogenous amino acid pool sizes in mouse eggs and preimplantation embryos. J Reprod Fertil 61: 387–393. 7193734 10.1530/jrf.0.0610387

[61] Satterfield MC, Gao H, Li X, Wu G, Johnson GA, et al (2010) Select nutrients and their associated transporters are increased in the ovine uterus following early progesterone administration. Biol Reprod 82: 224–231. 19696016 10.1095/biolreprod.109.076729

[62] Hong J, Lee E (2007) Intrafollicular amino acid concentration and the effect of amino acids in a defined maturation medium on porcine oocyte maturation, fertilization, and preimplantation development. Theriogenology 68: 728–735. 17658593 10.1016/j.theriogenology.2007.06.002

[63] Partridge RJ, Leese HJ (1996) Consumption of amino acids by bovine preimplantation embryos. Reprod Fertil Dev 8: 945–950. 8896028 10.1071/rd9960945

[64] Lamb VK, Leese HJ (1994) Uptake of a mixture of amino acids by mouse blastocysts. J Reprod Fertil 102: 169–175. 7799310 10.1530/jrf.0.1020169

[65] Spindle A (1995) Beneficial effects of taurine on mouse zygotes developing in protein-free culture medium. Theriogenology 44: 761–772. 16727773 10.1016/0093-691x(95)00275-d

[66] Suzuki C, Yoshioka K, Sakatani M, Takahashi M (2007) Glutamine and hypotaurine improves intracellular oxidative status and in vitro development of porcine preimplantation embryos. Zygote 15: 317–324. 17967211 10.1017/S0967199407004273

[67] Leese HJ, Hugentobler SA, Gray SM, Morris DG, Sturmey RG, et al (2008) Female reproductive tract fluids: composition, mechanism of formation and potential role in the developmental origins of health and disease. Reprod Fertil Dev 20: 1–8. 10.1071/rd07153 18154692

[68] VanWinkle LJ, Tesch JK, Shah A, Campione AL (2006) System B0,+ amino acid transport regulates the penetration stage of blastocyst implantation with possible long-term developmental consequences through adulthood. HumReprod Update 12: 145–57. 10.1093/humupd/dmi044 16251251

[69] Richards T, Wang F, Liu L, Baltz JM (2010) Rescue of postcompaction-stage mouse embryo development from hypertonicity by amino acid transporter substrates that may function as organic osmolytes. Biol Reprod 82: 769–777. 20018907 10.1095/biolreprod.109.081646

[70] Zhao X, Ma W, Das SK, Dey SK, Paria BC (2000) Blastocyst H(2) receptor is the target for uterine histamine in implantation in the mouse. Development 127: 2643–2651. 10821762 10.1242/dev.127.12.2643

[71] Groebner AE, Rubio-Aliaga I, Schulke K, Reichenbach HD, Daniel H, et al (2011) Increase of essential amino acids in the bovine uterine lumen during preimplantation development. Reproduction 141: 685–695. 21383026 10.1530/REP-10-0533

[72] Chisholm JC, Johnson MH, Warren PD, Fleming TP, Pickering SJ (1985) Developmental variability within and between mouse expanding blastocysts and their ICMs. J Embryol Exp Morphol 86: 311–336. 4031746

[73] Coan PM, Vaughan OR, McCarthy J, Mactier C, Burton GJ, et al (2011) Dietary composition programmes placental phenotype in mice. J Physiol 589: 3659–3670. 21624969 10.1113/jphysiol.2011.208629 PMC3167124

[74] Mitchell M, Schulz SL, Armstrong DT, Lane M (2009) Metabolic and mitochondrial dysfunction in early mouse embryos following maternal dietary protein intervention. Biol Reprod 80: 622–30. 19129514 10.1095/biolreprod.108.072595 PMC2849812

[75] Choo AY, Blenis J (2009) Not all substrates are treated equally: implications for mTOR, rapamycin-resistance and cancer therapy. Cell Cycle 8: 567–572. 19197153 10.4161/cc.8.4.7659

[76] Carling D, Thornton C, Woods A, Sanders MJ (2012) AMP-activated protein kinase: new regulation, new roles? Biochem J 445: 11–27. 22702974 10.1042/BJ20120546

[77] Langley SC, Jackson AA (1994) Increased systolic blood pressure in adult rats induced by fetal exposure to maternal low protein diets. Clin Sci (Lond) 86: 217–222. 8143432 10.1042/cs0860217

[78] Handyside AH, Hunter S (1984) A rapid procedure for visualising the inner cell mass and trophectoderm nuclei of mouse blastocysts in situ using polynucleotide-specific fluorochromes. J Exp Zool 231: 429–434. 6209359 10.1002/jez.1402310317

